# Using adalimumab serum concentration to choose a subsequent biological DMARD in rheumatoid arthritis patients failing adalimumab treatment (ADDORA-switch): study protocol for a fully blinded randomised superiority test-treatment trial

**DOI:** 10.1186/s13063-021-05358-7

**Published:** 2021-06-19

**Authors:** Maike H. M. Wientjes, Sadaf Atiqi, Gerrit Jan Wolbink, Michael T. Nurmohamed, Maarten Boers, Theo Rispens, Annick de Vries, Ronald F. van Vollenhoven, Bart J. F. van den Bemt, Alfons A. den Broeder

**Affiliations:** 1 Department of Rheumatology, Sint Maartenskliniek, PO box 9011, 6500 GM Nijmegen, the Netherlands; 2 Department of Rheumatology, Amsterdam Rheumatology and Immunology Center, Reade, Amsterdam, the Netherlands; 3 Biologics Lab, Sanquin Diagnostic Services, Sanquin, Amsterdam, the Netherlands; 4 Department of Epidemiology and Data Science, Amsterdam Rheumatology and Immunology Center, UMC Amsterdam, Vrije Universiteit, Amsterdam, the Netherlands; 5 Department of Rheumatology, Amsterdam Rheumatology and Immunology Center, UMC/Academic Medical Center, Amsterdam, the Netherlands; 6 Department of Pharmacy, Sint Maartenskliniek, Nijmegen, the Netherlands; 7 Department of Pharmacy, Radboud University Medical Center, Nijmegen, the Netherlands; 8 Department of Rheumatology, Radboud University Medical Center, Nijmegen, the Netherlands

**Keywords:** Rheumatoid arthritis, Adalimumab, Anti-TNF, Therapeutic drug monitoring, Drug concentration, Switching, Test-treatment trial, Design

## Abstract

**Background:**

A substantial proportion of rheumatoid arthritis (RA) patients discontinues treatment with tumour necrosis factor inhibitors (TNFi) due to inefficacy or intolerance. After the failure of treatment with a TNFi, treatment can be switched to another TNFi or a bDMARD with a different mode of action (non-TNFi). Measurement of serum drug concentrations and/or anti-drug antibodies (therapeutic drug monitoring (TDM)) may help to inform the choice for the next step. However, the clinical utility of TDM to guide switching has not been investigated in a randomised test-treatment study.

**Methods:**

ADDORA-switch is a 24-week, multi-centre, triple-blinded, superiority test-treatment randomised controlled trial. A total of 84 RA patients failing adalimumab treatment (treatment failure defined as DAS28-CRP > 2.9) will be randomised in a 1:1 ratio to a switching strategy to either TNFi or non-TNFi based on adalimumab serum trough level (intervention group) or random allocation (control group). The primary outcome is the between-group difference in mean time-weighted DAS28 over 24 weeks.

**Discussion:**

The trial design differs in many aspects from previously published and ongoing TDM studies and is considered the first blinded test-treatment trial using TDM in RA. Several choices in the design of this trial are described, and overarching principles regarding test-treatment trials and clinical utility of TDM are discussed in further detail.

**Trial registration:**

Dutch Trial Register NL8210. Registered on 3 December 2019 (CMO NL69841.091.19).

**Supplementary Information:**

The online version contains supplementary material available at 10.1186/s13063-021-05358-7.

## Background

Tumour necrosis factor inhibitors (TNFi) have improved treatment of rheumatoid arthritis (RA), but a proportion of patients discontinues treatment due to inefficacy or intolerance [1]. The 2019 European League Against Rheumatism (EULAR) recommendations for the management of RA advocate that any biologic agent including a subsequent TNFi can be used with on a group level equal chance for effect in case of non-response to a previous TNFi, based on a meta-analysis of three randomised controlled studies [2–6]. In addition, currently, no strong predictors for response to different types of biologic disease-modifying anti-rheumatic drugs (bDMARDs) in RA are available [7]. Therefore, after the failure of treatment with a TNFi, two approaches are viable with an equal chance of response: treatment with another TNFi (adalimumab, certolizumab, etanercept, golimumab, infliximab) or treatment with a bDMARD with a different mode of action (non-TNFi: abatacept, rituximab, sarilumab, tocilizumab).

However, it is hypothesised that therapeutic drug monitoring (measurement of drug concentrations and/or anti-drug antibodies, TDM) might help the clinician in choosing between treatment with another TNFi or treatment with a bDMARD with a different mode of action. In this study, we focus on the failure to adalimumab, a fully human monoclonal antibody TNFi that is one of the most frequently prescribed TNFi worldwide.

Failure to respond to adalimumab treatment can have multiple causes. First, some patients are not sensitive to TNF blockade and experience innate TNFi insensitivity. In these patients, switching to a non-TNFi is—at least conceptually—superior to starting a second TNFi. Secondly, approximately 30% of RA patients using adalimumab develop a substantial amount of antibodies against adalimumab (anti-drug antibodies (ADA)) that adversely affect pharmacokinetics, causing immediate (primary) or delayed (i.e. after the initial response, secondary) non-response [8–10]. Patients developing ADA may thus experience drug failure, but not necessarily class failure; they can still respond adequately to a second TNFi. This seems especially likely in secondary non-responders as they were primary TNFi responders; therefore, in these secondary non-responders, response rates to a second TNFi might be expected to be even higher than to a non-TNFi.

The above-mentioned hypothesis has been tested in multiple studies. Firstly, a systematic review addressing the above-mentioned hypothesis found three studies investigating TDM for prediction of response on a new bDMARD in case of inefficacy to the first bDMARD [11]. Two studies were performed in RA patients and one in a cohort of spondyloarthropathy patients [12–14]. Two studies showed that the development of ADA in patients who failed to respond to a first TNFi predicts a better clinical response to a second TNFi; one study concluded the same although this was not statistically significant. A study published more recently confirmed this, showing that RA patients with inadequate response to adalimumab, despite having sufficient adalimumab concentrations, benefit less from switching to etanercept than patients with low adalimumab concentrations [10]. These studies have some limitations, as the number of patients is limited, and test characteristics (sensitivity, specificity) were not mentioned. Additionally, as only switching from a TNFi to another TNFi is addressed, the predictive value of TDM for response to non-TNFi after failure to TNFi remains unclear. The latter is considered relevant to determine whether substantial ADA, impacting on pharmacokinetics, is able to differentially predict response to a second TNFi compared to a non-TNFi. A recent retrospective study by Ulijn et al. tried to address these issues by using larger patient numbers, assessing formal test characteristics, and including switchers to non-TNFi. This study did not show a predictive value of either ADA or randomly timed drug concentrations for obtaining a response to a second TNFi or non-TNFi [9]. This could have been due to the randomly timed sampling instead of trough level timed sampling; however, recent data suggest that results from both approaches are very comparable [15]. Of note, a recent review regarding the immunogenicity of TNFi in RA suggested that clinical effects of ADA depend on the amount of drug that is neutralised by ADA, and the amount of free drug present [16]. Even in the presence of ADA, drug concentrations can still be sufficient to reach clinical remission; therefore, measurement of serum drug concentrations is assumed to be more valid compared to ADA on its own.

In conclusion, although the use of TDM for the selection of the best subsequent DMARD in adalimumab failing RA patients seems promising, this has not yet been studied in a prospective intervention study. Therefore, we set up a randomised controlled test-treatment trial evaluating whether a switching strategy based on measurement of adalimumab trough serum concentration at the moment of failure is superior to random switching in reducing disease activity for RA patients failing adalimumab treatment with respect to disease control.

## Methods

### Trial design

**Fig. 1:**
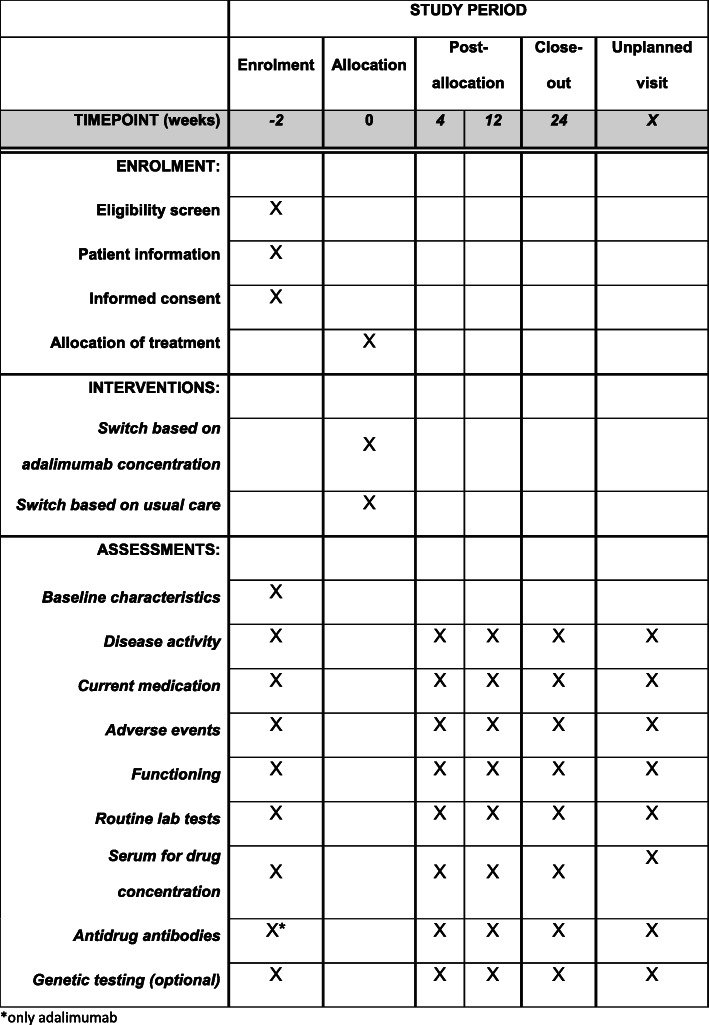
SPIRIT figure: trial visits and assessments

The ADDORA-switch is a multi-centre, triple-blinded, superiority test-treatment RCT in patients with RA starting another bDMARD after adalimumab failure (Fig. 1). The study is set up in a collaboration between Reade, the Sint Maartenskliniek and Sanquin Diagnostics and funded by ZonMw and Sanquin Diagnostics. The study started on 31 July 2020 and is expected to be performed in at least four departments of rheumatology of hospitals in the Netherlands: the Sint Maartenskliniek, Reade, Amsterdam UMC (location VUmc) and Reumazorg Zuid West Nederland. The study has received ethical review board approval (number NL69841.091.19) and has been registered (Dutch Trial Register NL8210). A data safety and monitoring board (DSMB) will be installed which reviews data on recruitment, efficacy, safety, protocol adherence, protocol updates and results of monitoring visits. Standard Protocol Items: Recommendation for Interventional Trials (SPIRIT) checklist is shown in Additional file 1.

### Objectives

The aim of this study is to evaluate whether a switching strategy based on adalimumab trough serum concentration at the moment of failure is superior to random switching in RA patients failing adalimumab treatment with regard to mean time-weighted disease activity score (DAS28-CRP) over 24 weeks. The secondary objectives are to compare the EULAR good response rates after 12 and 24 weeks, to compare the percentages of patients reaching low disease activity (DAS28-CRP < 2.9) or remission (DAS28-CRP < 2.4) after 24 weeks, to compare percentages of EULAR non-responders to the subsequent biological and to assess the number and severity of adverse events and the use of co-medication/rescue medication.

### Participants

Patients with rheumatoid arthritis (according to ACR 1987 and/or 2010 criteria and/or clinical diagnosis) who recently failed adalimumab treatment (defined as DAS28-CRP > 2.9) and are ≥ 16 years of age are eligible to participate in this study. Patients are included if they have received adalimumab (originator or biosimilar) for at least the last 10 weeks in standard dosing (40 mg subcutaneously every other week, either in monotherapy or combined with methotrexate or leflunomide) and if they are due to stop adalimumab due to lack of efficacy, either alone or combined with side effects. This particular adalimumab exposition (time and dose) was chosen to ensure sufficient exposure to adalimumab to infer true failure. At inclusion, patients still have to be on adalimumab treatment to assure valid measurement of adalimumab trough concentrations and anti-drug antibodies. Treatment with co-medication, including DMARDs and prednisone, is allowed at inclusion and during the study, as this maximises the generalisability of the results to the daily clinical practice.

Patients are excluded if they had been treated with another TNFi prior to adalimumab, as these patients risk randomisation to etanercept (being their third TNFi) and evidence suggests that changes of response are lower for treatment with a third TNFi [17, 18]. Treatment with other bDMARDs prior to adalimumab is allowed, but patients who have received all allowed non-TNFi options—abatacept, rituximab, sarilumab or tocilizumab—are excluded. Other exclusion criteria include contraindication to receive either etanercept or all allowed non-TNFi bDMARD (abatacept, rituximab, sarilumab or tocilizumab), life expectancy < 6 months, scheduled surgery or other pre-planned reasons for treatment discontinuation during follow-up.

### Patient recruitment

All eligible patients are selected and approached based on information from the electronic health record, according to the above-mentioned inclusion and exclusion criteria. The treating rheumatologist informs patients and asks them to participate in this study using a letter accompanied by the patient information sheet and the informed consent form. Patients receive this information 2 weeks prior to a planned outpatient clinic visit. Additionally, the researcher calls the patient to further explain the study, discuss the logistics of inclusion and answer the questions. If they have any additional questions, they can contact the local researcher or their treating rheumatologist. At the outpatient clinic visit, the study is discussed and informed consent is obtained. After informed consent is obtained, baseline data are collected and patients receive the allocated study medication. Patients will be recruited from four participating hospitals (the Sint Maartenskliniek, Reade, Amsterdam UMC (location VUmc) and Reumazorg Zuid West Nederland).

### Randomisation and blinding

**Fig. 2:**
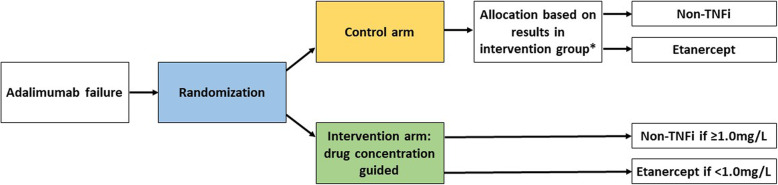
Flow diagram of the ADDORA-switch study. *Allocation of subsequent treatment in the control group is directly based on allocation in the intervention group. In the case of randomisation allocating more patients to the control group than currently allocated to the intervention group, these patients will be randomised in a ratio of 1:2 to etanercept or a non-TNFi

Eligible patients are randomised in a ratio of 1:1 to a switching strategy based on adalimumab concentration in addition to usual care (intervention group) or random switching strategy in addition to usual care (control group) (Fig. 2). Randomisation blocks are generated by an online programme and have variable block sizes (2 or 4 per block) to achieve the intended allocation ratio and to prevent the allocation from being predictable for the treating rheumatologist. The randomisation sequence is concealed for all trial participants and professionals (patients, physicians and assessors).

Subsequent treatment for the intervention group is based on adalimumab trough concentration. Patients with an adalimumab trough concentration below 1.0 mg/L (either with or without measurable anti-drug antibodies) receive another TNFi, etanercept, while patients with an adalimumab concentration of 1.0 mg/L or higher receive a non-TNFi bDMARD. The choice for a particular bDMARD is left up to the treating health care provider and maybe one of abatacept, rituximab, sarilumab or tocilizumab. In the control group, patients receive instead a second randomisation to either etanercept or the same choice of non-TNFi bDMARD (abatacept, rituximab, sarilumab or tocilizumab).

A somewhat complex allocation and blinding design is used to be able to perform this comparison with patients and physicians being fully blinded. A fully blinded design is very rarely used in a test-treatment or diagnostic study but is—when feasible—the optimal design as it prevents a behavioural change in patients, physicians and assessors (expectation bias, detection bias). Also, our design results in a comparison that is as fair as possible as the proportion of TNFi and non-TNFi given to patients is harmonised by group in order to prevent that between-group differences originate from an imbalance in allocation and effectiveness between TNFi or non-TNFi or co-DMARDs in these patients.

Firstly, we blind the primary allocation to intervention (TDM) or control group and the results from the testing for adalimumab concentrations. This is done by letting an independent researcher perform the primary allocation and by sending a concrete treatment advice instead of adalimumab concentration results to study physicians. In case a patient is randomised to intervention, based on the serum adalimumab concentration (see above), the physician receives an advice to give either etanercept or a non-TNFi bDMARD chosen by the treating physician. When a patient is randomised to control, serum adalimumab concentrations are not determined immediately and treatment is based on a secondary allocation and again sent to study physicians in the form of a treatment advice. This way, the health care provider only receives a treatment advice (‘etanercept’ or ‘non-TNFi bDMARD’), without knowing whether it is based on test result or chance.

We chose to standardise the control group as well, as preference policies regarding a second bDMARD after failure to adalimumab differ per centre. Several options were available for the secondary allocation (TNFi or non-TNFi in the control group). Ideally, this allocation would follow the same TNFi vs non-TNFi ratio as the intervention group. Therefore, a secondary randomisation with a ratio of 1:1 would not be optimal because previous data suggest that approximately 30% of patients have an adalimumab concentration below 1.0 mg/L [8–10]. A fixed 1:2 ratio TNFi vs non-TNFi would be better, but the proportion of patients with low adalimumab concentrations might be different in our population. Therefore, we chose to base the allocation of subsequent treatment in the control group directly on the allocation in the intervention group. When operationalised, this dependency means that when a patient in the intervention group is receiving etanercept, the subsequent patient randomised to the control group will also be assigned to etanercept. In case of temporary imbalance with an excess of patients allocated to the control group, these patients will be randomised in a ratio of 1:2 to etanercept or a non-TNFi, as this corresponds with expectations explained above.

### Interventions

In both groups, patients commence treatment as explained above and visit the clinic after 1, 3 and 6 months. Of note, bDMARD treatment (etanercept, abatacept, rituximab, sarilumab or tocilizumab) may be combined with continued methotrexate or leflunomide in registered dose, according to label and clinical practice. The dosing of the subsequent bDMARD is according to the authorised dose, except for rituximab, that also may be dosed 1 × 1000 mg or 2 × 500 mg instead of 2 × 1000 mg i.v [19].

At every visit, disease activity will be measured by a trained physician, and patients are treated to the target of DAS28-CRP at least below 2.9. In case of an increased or persistently high disease activity between visits, patients are encouraged to contact the clinic and an additional clinic visit is scheduled. Treatment with anti-rheumatic co-medication during the study is allowed and left to the discretion of the treating rheumatologist; however, we aim to keep co-DMARDs stable during follow-up. Changes in co-medication during follow-up of the study are allowed in case of flare (an increase of DAS28-CRP of > 1.2, or > 0.6 when current DAS28-CRP ≤ 2.9), adverse events, or for RA unrelated reasons. In case of active disease, bridging treatment is offered to patients, consisting of NSAIDs, methylprednisolone 120 mg i.m. (Depo-medrol®), triamcinolone i.m. 80 mg (Kenacort®) or oral prednisone (usually 15 mg and tapered in 2–6 weeks). In case of a persistent flare, the bDMARD can be switched to any other b/cs/tsDMARD treatment. All adverse events reported spontaneously by the subject or observed by the investigator or the physician will be recorded. To optimise protocol adherence, physicians are trained by the research team and researchers monitor adherence of the physicians and will—where needed—give feedback.

At the first visit (baseline visit, the patient is still using adalimumab), serum samples will be collected to directly analyse trough adalimumab concentrations and anti-adalimumab antibodies in intervention patients. Trough adalimumab concentrations will be measured instead of randomly timed concentrations, as literature shows more potential for trough concentrations compared to randomly timed concentrations [9, 12]. A trough concentration is defined as the serum concentration just prior to the next injection of adalimumab, with a margin of 3 days before the injection.

### Testing of serum drug concentrations and anti-drug antibodies

Trough serum adalimumab concentrations will be measured by enzyme-linked immunosorbent assay (ELISA). The detection limit of the assay is 0.001 mg/L [20]. A cut-off concentration of 1.0 mg/L was chosen based on the combination of results from previous studies [1, 10]. Anti-drug antibodies (ADA) will be measured using a radioimmuno assay (RIA); ADA will be expressed in arbitrary units (AU/mL). Patients were said to be positive for ADA if at one time point ADA were above the cut-off of 12 AU/mL (limit of detection [LoD]), ADA are quantifiable above 30 AU/mL (lower limit of quantification [LLOQ]). All testing will be performed by Sanquin Diagnostics, the Netherlands. The validation procedures and execution of the serum concentration and ADA assays for the adalimumab have been accredited by the RvA/CCKL (Dutch Accreditation Council/Dutch Accreditation Board for Medical Laboratories) according to the International Standardization Organization (ISO) guideline ISO15189.

### Outcomes

The primary outcome of this study is the between-group difference in mean time-weighted Disease Activity Score in 28 joints (DAS28-CRP) after 24 weeks. Time-weighted means taking into consideration not only the numerical level of a variable, but also the amount of time spent on it (trapezoidal rule). The DAS28-CRP is considered a valid, reliable and broadly accepted indicator of the clinical activity of RA. Since patients are treated to target, it is possible that at the end of the study, no difference in DAS28 scores is found between the two groups, while the flaring frequency might indeed be higher in one of the groups. This is why mean time-weighted DAS28 (MTW-DAS28) is chosen above the DAS28 score at the study end. A flare is defined as an increase of DAS28-CRP compared to DAS28-CRP at the former visit with > 1.2, or > 0.6 when current DAS28-CRP ≤ 2.9 [21]. Any switch of DMARD, as well as bridging treatment for more than 6 weeks, is considered treatment failure. In case of treatment failure, the patients will remain in the study, but the last measure of the DAS28-CRP and other outcomes before initiation of bridging/escape medication is carried forward (last observation carried forward). Lack of response is defined as no improvement by at most 3 months after the start of the subsequent biological or the absence of low disease activity by 6 months.

Secondary outcomes include the percentage of patients with good or moderate response according to the EULAR response criteria after 12 and 24 weeks, percentage of patients with minimal disease activity (DAS28-CRP < 2.9) after 24 weeks, percentage of non-responders to the subsequent biological after 24 weeks, number of flares after 24 weeks, number and severity of adverse events and use of co-medication/rescue medication.

### Sample size

The power calculation for this study is based on the superiority principle, since we expect better outcomes for patients in whom the choice of subsequent biological is based on serum drug concentrations. The primary outcome, MTW-DAS28, is expected to be 2.8 in patients with low disease activity. Based on the DRESS study and STRASS study, SD of the mean time-weighted DAS28 after 24 weeks of treatment is expected to be 0.9 [22, 23]. The difference in disease activity is defined as the change in MTW-DAS28 ≥ 0.6, as this is the measurement error of DAS28 and is considered a clinically relevant difference of clinical status in several studies [24–27]. With a two-sided 5% significance level, power of 80% and randomisation ratio 1:1, the sample size is 72 (36 per group). To allow for 15% dropout, the final sample size is 84 patients.

### Data analysis

Data is extracted from the electronic health records and anonymously entered and stored in the data management system Castor EDC. All statistical analyses will be performed with IBM SPSS version 23 or STATAIC 13. The researcher will be blinded for allocation of intervention and control. First, data will be analysed according to intention-to-treat (ITT) analysis in order to assess efficacy potentially influenced by second-order effects. Additionally, also a per-protocol analysis will be performed as this provides the most optimistic estimate of the true efficacy of an intervention. In case of treatment failure, the last measure of the DAS28-CRP before initiation of bridging/escape medication is carried forward (last observation carried forward). The number and reasons for dropout are reported to ensure internal validity.

Descriptive statistics of baseline characteristics will be provided with mean, standard deviation, median (p25–p75) or n (%) depending on data distribution. The size of differences in the primary outcome, mean time-weighted DAS28-CRP, between the two study arms will be calculated and presented. Additionally, the difference will be assessed with Student’s t-test. Pre-specified adjusted analyses are performed on the primary ITT outcome, adjusting for rheumatoid factor (RF)/anti-citrullinated protein antibodies (ACPA) status [28] and baseline DAS28-CRP [29]. A two-sided p-value < 0.05 is considered statistically significant. Secondary outcomes are compared with Fischer’s exact test, except for the number and severity of adverse events and the use of co-medication. Subgroup analyses will be performed to assess the differences in the effect of the intervention for patients experiencing primary (≤6 months) or secondary (> 6 months) failure to adalimumab. No interim analyses for safety, efficacy or futility will be performed.

## Discussion

The ADDORA-switch study will evaluate the additive value of measurement of adalimumab serum concentrations at the moment of failure to adalimumab in determining the subsequent biological in RA patients. The trial design differs in many aspects from previously published and ongoing TDM studies and can be considered the first test-treatment RCT using therapeutic drug monitoring in RA. Topics addressed in the design involve some interesting overarching principles we would like to discuss in further detail.

Firstly, the design of the ADDORA-switch is a rare example of a test-treatment trial in which a fully blinded design is possible. Blinding is considered very important in controlled trials, as it prevents unequal provision of care (performance bias) and biased assessment of the outcome (detection bias or expectation bias) [30]. According to a review by Ferrante di Ruffano, 76% of test-treatment trials do not use any type of blinding; patients and physicians were only blinded in 5 and 4%, respectively; and full blinding was exceptionally rare (2%) [31]. Not blinding trial participants will have consequences on the validity of trial results as produced results risk reflecting expectations of participants, clinicians and trialists, which is known to generally cause overestimated treatment effects. However, practical and ethical difficulties involved in blinding test-treatment trial participants (patients, care providers and assessors) are part of the reason why blinding in this type of trial is particularly rare. Remarkably, the ADDORA-switch has a fully blinded design which is possible due to multiple reasons related to the test and the treatment. First, blinding for conduction of the test is not necessary as it is considered ethical to conduct serum sampling in all patients. Also, as test results are solely known by independent researchers performing the randomisation, test outcomes remain unknown for all trial participants including clinicians. Lastly, it is a substantial benefit of our trial that treatment options are comparable for both groups and used in both groups; therefore, blinding treatment is not necessary.

Another point of interest considers operationalisation of TDM in current practice, which can be logistically rather challenging for several reasons. First, it is essential to obtain the blood sample at the correct time after dosing to assess the trough drug concentration; therefore, the patient’s current medication regimen has to be closely managed. Also, for interpretation, the concentration measurements and the sampling time need to be considered in relation to drug dose and dosage history. Additionally, laboratory turnaround time—the time between ordering a test and reporting of results—causes that clinicians cannot directly prescribe the new drug during consultation. Prescription of a subsequent drug based on TDM is therefore more time-consuming compared to the current practice. The last challenge is associated with the costs of additional testing. Especially when samples have to be analysed externally, analysis can be costly. Therefore, the cost-effectiveness of TDM in clinical practice has to be investigated in more detail.

Lastly, the effects of additional testing on a background of current state of art treatment strategies should be evaluated. The use of a treat-to-target strategy, which defines a treatment target and applies tight control to reach this target, is widely embedded in the treatment of RA. Following this, high disease activity is treated aggressively until patients reach and maintain remission or low disease activity, and this results in a second-order effect that might negate positive effects of TDM. Also, part of the patients will switch to another drug that would have been in line with TDM-guided switching already. We hypothesise that therapeutic drug monitoring nevertheless might contribute to channelling the right patients to the right drug, thus reducing overall disease activity and lowering the incidence of subsequent bDMARD switching.

### Trial status

The trial started with the recruitment on 31 July 2020 and is currently recruiting. Recruitment is estimated to be finished in July 2022. Protocol version 2.3, 30-10-2020.

## Supplementary Information

**Additional file 1.** SPIRIT 2013 Checklist.

## Data Availability

The datasets collected during the current study are available from the corresponding author on reasonable request.

## Abbreviations

ACPA: Anti-citrullinated protein antibodies
ACR: American College of Rheumatology
ADA: Anti-drug antibodies
ADDORA: Adalimumab dose optimization in Rheumatoid Arthritis
ADDORA-switch: Using adalimumab serum concentration to choose a subsequent biological DMARD in rheumatoid arthritis patients failing adalimumab treatment
AU: Arbitrary units
bDMARD: Biologic disease-modifying anti-rheumatic drug
CDAI: Crohn’s Disease Activity Index
CMO: Commissie Mensgebonden Onderzoek
CRP: C-reactive protein
DAS28: Disease Activity Score based on 28 joints
DMARD: Disease-modifying anti-rheumatic drug
ELISA: Enzyme-linked immunosorbent assay
EULAR: European League Against Rheumatism
TDM: Therapeutic drug monitoring
TNFi: Tumour necrosis factor inhibitors
ISO: International Standardization Organization
LoD: Limit of detection
LLOQ: Lower limit of quantification
MTW-DAS28: Mean time-weighted disease activity score over 28 joints
RA: Rheumatoid arthritis
RAPID-3: Routine Assessment of Patient Index Data 3
RCT: Randomised controlled trial
RIA: Radioimmuno assay
SDAI: Simple Disease Activity Index
sHAQ: Scleroderma Health Assessment Questionnaire
tsDMARD: Targeted synthetic disease-modifying anti-rheumatic drug
VAS: Visual analogue scale
